# Validation of motion perception of briefly displayed images using a tablet

**DOI:** 10.1038/s41598-018-34466-9

**Published:** 2018-10-30

**Authors:** Daniel Linares, Rafael Marin-Campos, Josep Dalmau, Albert Compte

**Affiliations:** 1 Institut d’Investigacions Biomèdiques August Pi i Sunyer (IDIBAPS), Barcelona, Spain; 2 Hospital Clínic, University of Barcelona, Barcelona, Spain; 3 Centro de Investigación Biomédica en Red de Enfermedades Raras (CIBERER), Barcelona, Spain; 4 Catalan Institution for Research and Advanced Studies (ICREA), Barcelona, Spain; 5 Department of Neurology, University of Pennsylvania, Philadelphia, PA USA

## Abstract

Motion perception of briefly displayed images has been reported to be abnormal in clinical populations afflicted with schizophrenia, major depression, autism, Alzheimer’s disease and epilepsy. These abnormalities have been measured using CRT monitors connected to a computer. Given that the use of this experimental set-up in clinical environments can be difficult, we tested whether motion perception of briefly displayed images could also be measured using a tablet. For 13 participants, we found similar estimates of motion discrimination on a tablet and a CRT. This validates a tablet to measure motion perception of briefly displayed images.

## Introduction

Abnormal motion perception of briefly displayed images has been reported in clinical populations afflicted with schizophrenia^1^, major depression^2,3^, autism^4,5^, Alzheimer’s disease^6^ and epilepsy^7^. For example, in schizophrenia the typical impairment in motion discriminability that occurs when a brief image increases size (perceptual surround suppression) is attenuated^1^.

Motion perception of brief images in clinical populations has been measured using CRT monitors^1–4,6,7^ or DLP projectors^5^ connected to a computer. These experimental setups display images accurately and precisely^8–10^, and at a high temporal rate of 100 Hz^6^ or 120 Hz^1–5,7^. But, installing, keeping and using them in clinical environments—such as examination or hospital rooms—can be difficult.

Mobile devices like tablets are more convenient instruments to measure motion perception in clinical environments. These devices, however, incorporate LCD screens, which in comparison to CRT monitors display images with worse spatiotemporal properties and at a lower temporal rate, typically of 60 Hz^8–10^. Nevertheless, the characteristics of the screen of a mobile device could be good enough to measure motion perception of brief images reliably. We test this idea here comparing motion perception of brief images using a conventional CRT running at 120 Hz and a tablet running at a fixed frame rate of 60 Hz.

## Results

**Figure 1:**
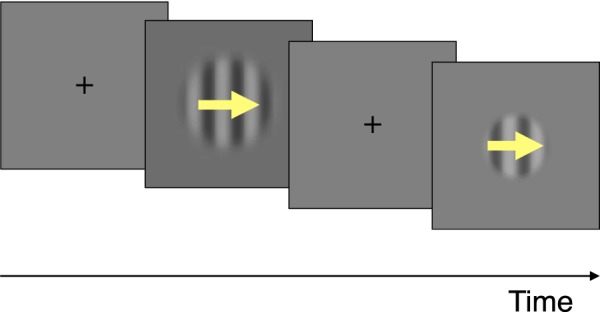
Illustration of two trials of the perceptual test to measure motion discrimination of briefly displayed images. Two sizes were used (see Methods): small (1 degree of visual angle) and large (4 degrees).

On each trial (Fig. 1), a briefly-presented grating (small or large) drifted to the left or to the right (the direction was chosen at random) and the participant reported the perceived direction. Participants performed half of the trials on a CRT and half on a tablet.

**Figure 2:**
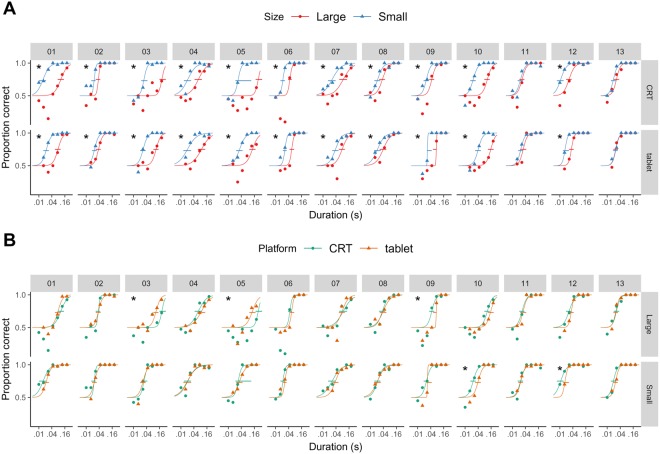
(**A**) Motion discrimination performance in 13 participants. The small horizontal segments represent the 99% bootstrap confidence intervals for the threshold. The threshold is represented by the intersection of the confidence interval with the psychometric function. An asterisk on the top-left of each panel indicates that the threshold is significantly different for the two sizes of the grating. (**B**) The data on (**A**) replotted to better visualize the differences between platforms. An asterisk on the top-left of each panel indicates that the threshold is significantly different for the two platforms.

Figure 2A shows, for each participant, the proportion of correct direction discriminations as a function of the duration of the grating, its size and whether the participant performed the task on a CRT or a tablet. For each participant and platform (CRT or tablet), we conjointly fitted two logistic psychometric functions—one for each size— to the proportion of correct discriminations as a function of the logarithm of grating duration. These two psychometric functions shared the slope parameter, as including independent slopes did not improve significantly the fit (likelihood ratio test, P > 0.01) for any participant except for participant 11 conducting the test on the CRT (likelihood ratio test, D = 14, P = 2 × 10^−4^).

To assess the goodness of fit, we calculated the deviance^11,12^. From the 26 fits (13 participants × 2 platforms), the deviance was not significant (P > 0.01, the P was calculated using bootstrap^12^) in 19. From the 7 fits with significant deviances, several include proportions for short durations below chance level 0.5 (for example, participants 1, 6 and 9 for the CRT). This systematic error to report the opposite motion direction that occurs in some participants for the shortest durations might be related to the reversals that have been described for very briefly displayed gratings^13^. We hypothesize that these reversals could be caused by participants reporting the motion direction of the afterimage^14^ instead of the motion direction of the stimulus. Indeed, it has been shown that for very briefly displayed gratings the motion direction of the afterimage is easier to discriminate than the stimulus that produces it^14^. It is unclear, however, why these reversals seem to occur only for the CRT. We think that more research is needed to understand this phenomenon.

To summarise motion discrimination, for each psychometric function, we calculated the duration threshold as the duration for which the participant responded correctly 75% of the times. We found that except for participant 11 and 13, for whom the stimulus size did not affect discrimination, the threshold duration needed to discriminate direction was longer for the large grating than for the small grating (bootstrap, see Methods, Fig. 2A), which indicates that motion discrimination was more difficult for large stimuli and replicates the phenomenon of perceptual surround suppression^15^. We also found that the thresholds estimated from trials of the first block were very similar to the thresholds estimated from the trials of the second block (see Methods), which indicates that both platforms were reliable (Supplementary Figure S1).

To check whether the estimated thresholds were sensible for the 7 fits that showed significant deviances (see above), we also estimated the thresholds for this data using a more flexible nonparametric model that better captured the nonlinearities at short durations using the *modelfree* package^16^. We found very similar thresholds to the ones found using the logistic model (Supplementary Figure S2).

**Figure 3:**
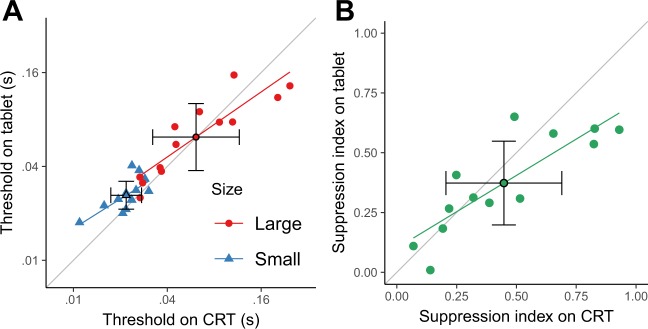
(**A**) The thresholds measured on the tablet against the thresholds measured on the CRT. (**B**) The suppression index measured on the tablet against the suppression index measured on the CRT. In (**A**) and (**B**) the symbols with black contours represent the geometric means and the error bars the 99% t-based confidence intervals.

Figure 2B replots the data of Fig. 2A, but directly comparing motion discrimination for the two platforms in individual panels. Except for three participants (3, 5 and 9) for the large grating and two participants (10 and 11) for the small grating, there were no differences between thresholds measured using the CRT and the tablet (bootstrap, see Methods). The correlation between the thresholds (in log units) measured on the CRT and the tablet was large and significant for the small grating (Fig. 3A; Pearson correlation, r_11_ = 0.69, P = 0.009) and for the large grating (Pearson correlation, r_11_ = 0.89, P = 4 × 10^−5^). Across participants, a two-way repeated measures ANOVA indicated a significant effect of size (F_1,12_ = 39; P = 4 × 10^−5^), no effect of platform (F_1,12_ = 2.5; P = 0.1) and no interaction of size by platform (F_1,12_ = 2.9; P = 0.1).

Figure 3B shows the suppression index—log_10_ (threshold for the large grating) −log_10_ (threshold for the small grating)—measured on the CRT against the suppression index measured on the tablet paired by participant. The correlation was large and significant (Pearson correlation, r_11_ = 0.84, P = 3 × 10^−4^). Across participants, the surround suppression index measured on the tablet (0.37) was not significantly different from that measured on the CRT (0.45; t_12_ = 1.7, P = 0.1).

## Discussion

We found similar duration thresholds and surround suppression indices for motion direction discrimination of brief gratings on a tablet and a CRT. This result validates the tablet to measure motion discrimination for this type of moving stimuli, as a CRT is the conventional experimental set-up to measure it^1–4,6,7,15^. We think that the validity of the tablet is important because motion discrimination of brief gratings have been shown to be altered in several neuropsychiatric conditions^1–7^. A tablet, by facilitating the measurement of this type of moving stimulus in clinical environments, will make it easier to collect large data samples, which could be important to establish the reproducibility of the findings and to test the influence of parametric changes of the stimuli.

We used an iPad instead of other tablet devices because this platform is readily available and it has a relatively small number of available models. This facilitates that the tablet application displays about the same stimuli in different models. Our application, for example, detects the iPad model and adapts the input size, input luminance and number of displayed frames in the code in a way that the size, luminance and duration of the grating displayed on the screen is about the same independently of the iPad model (in this study, we used just one model—the iPad 2017, but we also tested the flexibility of the application on the iPad Pro 1st generation running at 60 and 120 Hz). There are not many studies validating or using applications in tablet devices to perform perceptual tests, but the few reported in the literature also chose the iPad as platform^17–21^. One of these applications implements a test to measure the contrast sensitivity function^17^ and has been successfully used in patients who experienced extended early-onset blindness^18^. Another application implements a more general framework to display visual stimulation^19^ and has been successfully used to measure center-surround contrast suppression in patients with migraine^20^. These applications implement perceptual tests that display static stimuli. To our knowledge, our application is the first one to implement a valid perceptual test for dynamic stimuli.

To be able to assess perceptual surround suppression, we measured motion discrimination for small and large gratings. We matched the stimulus parameters to those in a previous study measuring perceptual surround suppression in patients with schizophrenia^1^. Across participants, we found an index of suppression of about 0.4, which is about the same that this previous study found in the group of healthy participants.

Interestingly, a previous study using stimuli like the ones we used showed that perceptual surround suppression for motion strongly correlates with intelligence^22^. A recent study also found this correlation, but of weaker magnitude^23^. Another recent study using a large sample^24^, however, could not replicate this finding. It has been suggested that a possible explanation of these conflicting findings is that in the large sample study, perceptual surround suppression was measured using an LCD monitor instead of a CRT or a DLP projector^23,24^. As tablets incorporate LCD screens, our results suggest that the conflicting findings might have another cause.

For all the participants of our sample, we were able to estimate motion discrimination thresholds using a tablet running at 60 Hz. This frame rate, however, could be too slow to measure motion discrimination for participants with very good motion sensitivity. This problem might be solved using the tablets like the iPad Pro running at 120 Hz.

Tablets and smartphones are mobile devices with a powerful hardware that can be used to implement a variety of behavioral tests and can revolutionize data acquisition in behavioral sciences^25^. An increasing number of studies are validating its use to administer cognitive^26–30^ and perceptual tests^17–20^. Here, we present a valid perceptual test to measure motion discrimination in a tablet device.

## Methods

### Participants

The study was approved by the ethical committee of the Hospital Clinic of Barcelona and followed the requirements of the Helsinki convention. Thirteen healthy participants (8 females) with a mean age of 28 years (standard deviation: 6 years) participated. They reported normal or corrected-to-normal visual acuity and did not know the hypothesis of the experiments. Informed consent was obtained for all participants.

### Stimuli

The stimuli displayed on the CRT (Philips 109 P; 19 inches, 800 × 600) were generated using PsychoPy^31^. The stimuli displayed on the iPad (iPad 2017; 9.7 inches, 2048 × 1536 pixels, GPU PowerVR GT7600) were generated using a custom application developed natively for iOS in XCode, an integrated development environment (IDE) for developing software using the programming language Swift. To optimize the performance, the functions that draw the stimuli on the screen were written in Metal, a low-level hardware-accelerated 3D graphic and computer shader application programming interface (API) that uses the graphics processing unit (GPU) of the device to perform the calculations in parallel.

To compute RGB values in real time in a screen with a refresh rate of 60 Hz, the computation should last less than 16.66 ms—the duration of a frame. Using Metal, the computation time was systematically below 4 ms (this calculation was performed using a debug session of Xcode, the GPU Frame Capture validation tool and the Metal API validation tool), which is significantly shorter than the duration of one frame. Displaying visual stimulation on real time is useful in vision science because it is not necessary to create look-up tables in advance.

Before testing, using an oscilloscope (Tektronix TDS 1012) and a photodiode, we verified that both platforms displayed square wave luminance profiles of 1, 2, 3 and 4 frames for the exact number of frames without dropping any. We also checked that the luminance profiles were stable across presentations. As has been described elsewhere^9^, we found that the luminance profile was more transient for CRT than for the tablet. To match the luminance of the two platforms, we used a photometer (Datacolor Spyder 5 Express).

The main stimuli were sinusoidal gratings (0.42 Michelson contrast) of 1 cycle per degree (of visual angle) drifting at 4 degrees/s with a Gaussian envelope of standard deviation of 0.5 degrees for the small grating and 2 degrees for the large grating. They were displayed in the center of the screen. On each trial, the initial phase of the grating was chosen randomly from a range of 5 values (0, 72, 144, 216 and 288°). The luminance of the background was 32 cd/m^2^. The screens were gamma-corrected using a photometer.

### Procedure

In some blocks, participants performed the perceptual test facing a CRT monitor. In other blocks, they performed the test holding the tablet on their lap. We controlled the viewing distance to be about 57 cm. To set up the viewing distance, at the beginning of each block the experimenter used a ruler to measure the distance from the eyes to the screen and asked the participant to change position (move the chair closer or away from the CRT or move the arms holding the tablet) until the distance was about 57 cm. Once the participant told us that she was in a comfortable position, we asked her to hold that position for the whole block. The experimenter was in the same experimental room controlling that the participant did not change position. Each participant performed 2 blocks on the CRT and 2 blocks on the tablet alternating between platforms (half of the participants started the test on the CRT and the other half on the tablet). We asked participants to look at the center of the screens during the tests. The testing was performed in a room with normal fluorescent lighting.

Each trial (Fig. 1) started with the presentation of a cross for 0.3 s. Then, a grating moving to the left or to the right (chosen at random on each trial) was presented. The duration of the grating was controlled using a temporal Gaussian envelope for the contrast of the grating and was chosen at random on each trial from a range of 7 logarithmically spaced durations starting at 0.01 s and finishing at 0.2 s; these durations were defined as 2 times the standard deviation of the Gaussian envelope. The peak of the Gaussian envelope occurred 0.3 s after the offset of the cross.

From the 7 durations tested, we decided not to include in the analysis the responses to the shortest stimuli displayed on the tablet for the following reason. The described temporal Gaussian envelope effectively displays the stimulus of the shortest duration (2 × standard deviation = 0.01 s) for 3 frames on the CRT (running at 120 Hz), but would display the stimulus for only 1 frame on the tablet given the smaller frame rate (60 Hz). Because motion cannot be defined with a single frame, we decided, for the shortest stimulus on the tablet, to displace the peak of the Gaussian envelope by half a frame. This effectively displayed the shortest stimulus on the tablet for 2 frames with a contrast smaller than the peak contrast of the shortest stimulus on the CRT, but higher than the contrast displayed in the first and third frame on the CRT. Although not very apparent on individual results, pulling the data across participants we realized that for the tablet performance was significantly higher for the shortest duration than for the second shortest duration. This abnormal result suggests that the workaround that we tried of displacing the peak of the Gaussian envelope in the tablet is not appropriate. For this reason, we decided to exclude from the analysis the responses to the shortest stimuli displayed on the tablet. This exclusion has a negligible impact on the threshold calculation because for this very short duration the proportion of correct responses was very close to chance.

Participants reported the perceived direction of motion using the arrow keys of a keyboard for the blocks performed on the CRT and tapping the left and the right part of the touchscreen for the blocks performed on the tablet. Feedback was not provided. The next trial started 0.3 s after the response.

Each block consisted of two parts of 140 trials (7 durations × 2 sizes × 2 directions × 5 initial phases, all randomly interleaved) with a short pause between parts of about one minute or less.

### Analysis

The data and the code to do the statistical analysis and create the figures is available at https://github.com/danilinares/2018LinaresMarinDalmauCompte. Psychometric functions were logistic functions with 0 lapse rate and 0.5 guess rate estimated by reweighted least squares using the function *glm* from the R software and the logit link function provided by the R package *psyphy*^32^. After submitting the study, we cross-validated the results using an updated version of the R package *quickpsy*^33^ that fits psychometric functions that share parameters using direct maximization of the likelihood (the code can be found in the repository above). The 99% confidence intervals for the thresholds were calculated using parametric bootstrap for 2000 bootstrap samples. To assess for each participant whether the thresholds for two different conditions were significantly different, we subtracted the bootstrapped thresholds for each condition (2000 pairs) and assessed whether the 0.5% and 99.5% percentiles of the distribution of differences contained 0.

## Electronic supplementary material

SUPPLEMENTARY FIGURES
