# Supplementary material for: Validation of motion perception of briefly displayed images using a tablet
Source: Sci Rep. 2018 Oct 30;8:16056. doi: 10.1038/s41598-018-34466-9 (PMC6207664; doi:10.1038/s41598-018-34466-9)
Supplement: Supplementary file 1 — SUPPLEMENTARY FIGURES [file 41598_2018_34466_MOESM1_ESM.pdf]

Validation of motion perception of briefly displayed images using a tablet

Daniel Linares[a,*], Rafael Marin[a], Josep Dalmau[a, b], Albert Compte[a]

[a] Institut d'Investigacions Biomèdiques August Pi i Sunyer (IDIBAPS), Barcelona, Spain.

[b] Hospital Clínic, University of Barcelona, Barcelona, Spain.
Centro de Investigación Biomédica en Red de Enfermedades Raras (CIBERER).
Department of Neurology, University of Pennsylvania, Philadelphia, PA, USA.
Catalan Institution for Research and Advanced Studies (ICREA), Barcelona, Spain.

[*] Corresponding author (danilinares@gmail.com)

SUPPLEMENTARY FIGURES

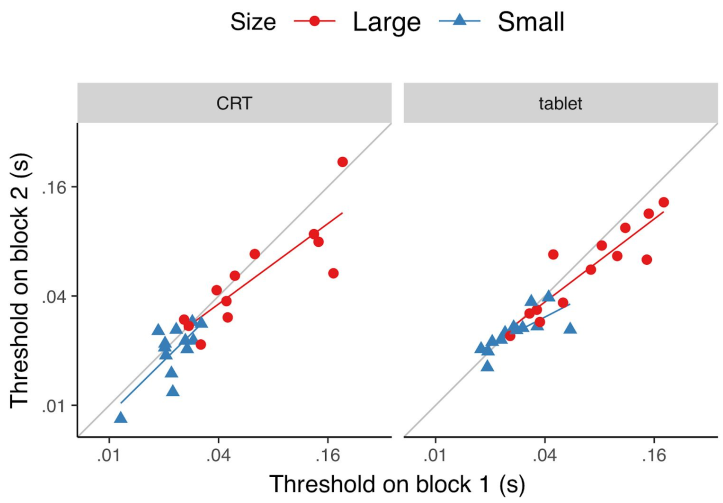

Supplementary Figure S1. The thresholds measured on the first block of trials against the thresholds measured on the second block of trials for the two platforms. To calculate the thresholds, for each participant, platform and size, we conjointly fitted two psychometric functions, one for each block, that shared the slope parameter. The thresholds for participant 5 (for the CRT and for large size) are not included because the psychometric function was flat. The Pearson correlations are the following: CRT and large condition (r_{10} = 0.85, P = 5 x 10^{-4}), CRT and small (r_{11} = 0.71, P = 0.006), iPad an large (r_{11} = 0.90, P = 2 x 10^{-5}), iPad and small (r_{11} = 0.73, P = 0.005).

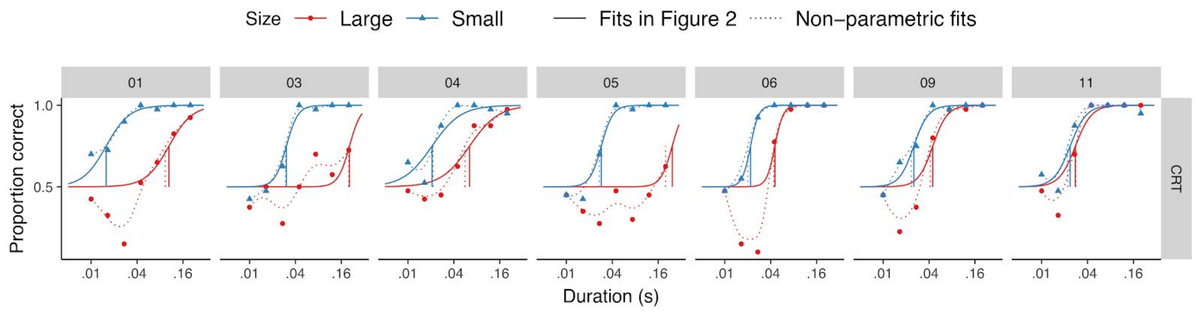

Supplementary Figure S2. Results from Figure 2A for the data with significant deviances in the goodness-of-fit test adding a nonparametric psychometric function fit. The vertical segments indicate the thresholds.
